# Early introduction of fish decreases the risk of eczema in infants

**DOI:** 10.1136/adc.2008.140418

**Published:** 2008-09-25

**Authors:** B Alm, N Åberg, L Erdes, P Möllborg, R Pettersson, S G Norvenius, E Goksör, G Wennergren

**Affiliations:** 1 Department of Paediatrics, University of Gothenburg, Queen Silvia Children’s Hospital, Gothenburg, Sweden; 2 Central Infant Welfare Unit, Uddevalla Hospital, Uddevalla, Sweden; 3 Paediatric Outpatient Clinic, Skene Hospital, Skene, Sweden; 4 Department of Paediatrics, Skaraborg Hospital, Skövde, Sweden

## Abstract

**Background:**

Atopic eczema in infants has increased in western societies. Environmental factors and the introduction of food may affect the risk of eczema.

**Aims:**

To investigate the prevalence of eczema among infants in western Sweden, describe patterns of food introduction and assess risk factors for eczema at 1 year of age.

**Methods:**

Data were obtained from a prospective, longitudinal cohort study of infants born in western Sweden in 2003; 8176 families were randomly selected and, 6 months after the infant’s birth, were invited to participate and received questionnaires. A second questionnaire was sent out when the infants were 12 months old. Both questionnaires were completed and medical birth register data were obtained for 4921 infants (60.2% of the selected population).

**Results:**

At 1 year of age, 20.9% of the infants had previous or current eczema. Median age at onset was 4 months. In multivariable analysis, familial occurrence of eczema, especially in siblings (OR 1.87; 95% confidence interval (CI) 1.50 to 2.33) or the mother (OR 1.54; 95% CI 1.30 to 1.84), remained an independent risk factor. Introducing fish before 9 months of age (OR 0.76; 95% CI 0.62 to 0.94) and having a bird in the home (OR 0.35; 95% CI 0.17 to 0.75) were beneficial.

**Conclusions:**

One in five infants suffer from eczema during the first year of life. Familial eczema increased the risk, while early fish introduction and bird keeping decreased it. Breast feeding and time of milk and egg introduction did not affect the risk.

Eczema in infants is a common health problem throughout the world, although the incidence varies.^1^ However, heredity is known to exert a major influence,^2^ and food and the period of food introduction may have an effect on age at onset and the severity of the disease.

There has been a substantial increase in the prevalence of allergic diseases in recent decades,^3^ ^4^ but the reasons for this are still unknown despite intensive research. The low prevalence of allergy in Eastern Europe was thought to be due to a difference in intestinal flora.^5^ In addition, data from experimental animal studies suggest that early contact with allergens might provide protection from subsequent sensitisation,^6^ which is also supported by epidemiological findings.^7^ However, other studies report different results.^8^

There is consensus that parental tobacco smoking plays an important role in the occurrence of early wheezing, but the influence on eczema is disputed.^9^^–^11 Dairy products,^12^ omega-3 fatty acids^13^ and a diet containing lactobacilli^14^ have been suggested to be protective factors against the development of atopy.

The aims of this study were to investigate the current prevalence of eczema among infants in western Sweden, to describe current patterns of food introduction and to assess the risk factors for eczema at 1 year of age.

## METHODS

“Infants of Western Sweden” is an ongoing study in western Sweden. The region has 1.5 million inhabitants (one sixth of the Swedish population) and consists of urban, rural and coastal areas. Gothenburg, with 500 000 inhabitants, is the largest city.

From the total birth cohort of 16 682 infants born in the region in 2003, 8176 families (50%) were randomly selected.^15^ When the infant was 6 months of age, the family was sent an invitation and a questionnaire (Q1) which sought detailed information on the family, environment, perinatal history, tobacco, breast feeding, food introduction and diseases in the first year, with special reference to allergic diseases. Families willing to participate returned written informed consent together with the questionnaire. These families were then sent a second questionnaire (Q2) when the child was 12 months of age. Q1 was sent out to 8176 families and completed by 5605, giving a response rate of 68.5%. Of these 5605 families, 4941 also completed Q2 (88.2% of those who answered Q1). Medical birth register data were available for 4921 (99.6%) of these 4941 (60.2% of the originally selected population). Details relating to the questionnaires have previously been published.^16^ ^17^

The question on eczema was: “Has your child had eczema? (y/n)”, while that on food allergy was: “Has your child a diagnosis of food allergy from a doctor? (y/n)”. Food frequency data were collected at 6 and 12 months.

In the statistical analysis of eczema, 2×2 tables with the χ^2^ test and binary logistic regression were used and risks were estimated using odds ratios (OR) with 95% confidence intervals (CI). Factors that were significant with a p value of <0.01 in the univariable analysis were then analysed in a multivariable model. Secondary (disease-associated) variables were excluded and further adjustment was carried out for “atopic reasons” for not having furry animals in the home (manifest allergy or fear of allergy), plus cow’s milk allergy. The SPSS statistical package v 15.0 was used for calculations.^18^

The study was approved by the ethics committee at the University of Gothenburg.

## RESULTS

### Eczema

At 6 months of age, 13.9% of families said that their infant had previous or current eczema, while the corresponding figure at 12 months was 20.9% (table 1). The median age at onset was 4 months (mean age 5.4 months).

**Table 1:** Reported prevalence of eczema, diagnosed food allergy, pet keeping and dietary factors in infants in western Sweden in 2004–2005: the Infants of Western Sweden Study

	n (%)
Eczema (n = 4953)	
At 6 months	692 (13.9)
At 12 months	1033 (20.9)
Food allergy (n = 4944)	
Any food allergy	241 (4.9)
Cow’s milk	194 (3.9)
Eggs	126 (2.5)
Fruit or vegetables	41 (0.83)
Cereals	18 (0.36)
Fish	14 (0.28)
Soy protein	12 (0.24)
Peanuts	10 (0.20)
Peas	9 (0.18)
Nuts or almonds	8 (0.16)
Pets in the home (n = 4987)	
Cat	1076 (21.6)
Dog	729 (14.6)
Rodent	162 (3.2)
Bird	113 (2.3)
Main type of fat on bread (n = 4893)	
Butter	3488 (71.3)
Margarine	1164 (23.8)
Nil	241 (4.9)
Frequency of lactic acid (yoghurt, fermented vegetables,	
etc) in the diet (n = 4929)	
3+ Times/week	2497 (50.7)
1–3 Times/week	1439 (29.2)
1–3 Times/month	531 (10.8)
Occasionally	161 (3.3)
Never	301 (6.1)
Frequency of fish consumption (n = 4953)	
3+ Times a week	250 (5.0)
1–3 Times a week	2995 (60.5)
1–3 Times a month	1296 (26.2)
A few times a year	217 (4.4)
Never	195 (3.9)
Type of fish usually consumed (n = 4473)	
Lean fish (cod, haddock)	3516 (78.6)
Salmon	772 (17.3)
Flatfish	140 (3.1)
Mackerel or herring	45 (1.0)

### Food allergy

Food allergy had been diagnosed by a physician in 4.9% of the study population (table 1).

### Furry pets

Many families had furry pets. The most common was a cat (21.6%), followed by a dog (14.6%) and rodents (3.2%). The keeping of pets was more common in rural than in urban areas. For example, dogs were kept by 8.4% of the households in the city of Gothenburg, while the figures were 25.8% and 20.7% in the counties of Dalsland and Skaraborg, respectively.

### Breast feeding and food introduction

**Figure 1:**
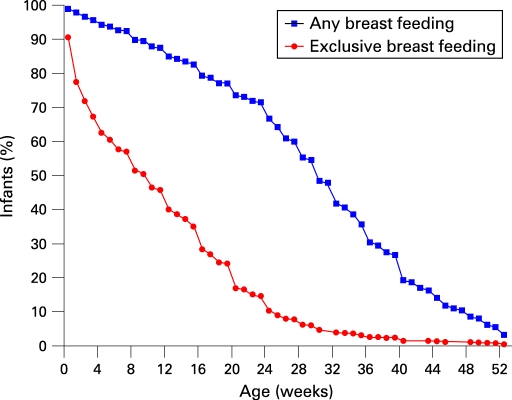
Prevalence of exclusive breast feeding and any breast feeding during the first year of life in western Sweden, 2003–2004.

**Figure 2:**
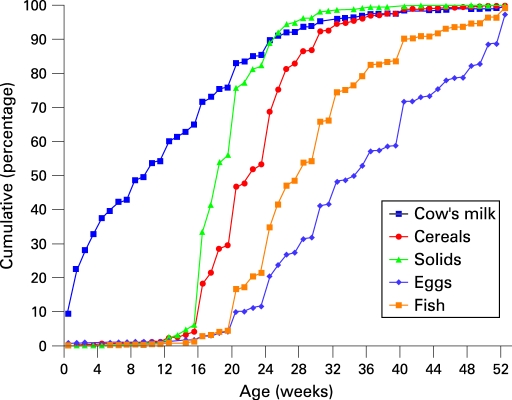
Food habits among infants: introduction of cow’s milk protein, cereals, solids, eggs and fish in western Sweden, 2003–2004.

Breast feeding and timing of the introduction of cow’s milk protein, cereals and solids are described in figs 1 and 2. At the age of 16 weeks, more than 80% of the infants were breast fed, while one third were exclusively breast fed (no cow’s milk protein introduced) (fig 1). At the age of 6 months, two thirds were still breast fed and 10% were exclusively breast fed. At about 30 weeks, 50% of the infants had been weaned (fig 1).

The median age for the introduction of cow’s milk protein was 9 weeks, compared with 18 weeks for the introduction of solids and 22 weeks for cereals (fig 2).

Fish was introduced at a median age of 7 months and eggs at a median age of 9 months (fig 2).

Butterfat was used on bread by 71.3% of the families at 12 months, while 50.7% consumed fermented dairy products (table 1) more than three times a week. When they ate fish, 18.3% mainly consumed fat fish such as salmon.

### Risk factors for eczema at 1 year

#### Univariable analysis

The variables significant at p<0.01 are presented in table 2. The non-significant variables with a p value between 0.01 and 0.05 as well as those with a p value ⩾0.05 are listed in the footnote.

**Table 2:** Prevalence of significant (p<0.01) risk factors for eczema at 1 year of age in the univariable analysis: the Infants of Western Sweden Study

Risk factor	Eczema	No eczema	OR (95% CI)	p Value
	n (%)	n (%)		
Paternal asthma	108 (10.5)	280 (7.2)	1.51 (1.20 to 1.91)	0.001
Maternal rhinoconjunctivitis	320 (31.3)	833 (21.5)	1.66 (1.42 to 1.93)	<0.001
Paternal rhinoconjunctivitis	287 (28.0)	876 (22.6)	1.33 (1.14 to 1.56)	<0.001
Sibling with rhinoconjunctivitis	68 (6.6)	179 (4.6)	1.47 (1.10 to 1.96)	0.009
Maternal eczema	392 (38.3)	917 (3.7)	2.00 (1.73 to 2.31)	<0.001
Paternal eczema	214 (20.9)	569 (14.7)	1.53 (1.29 to 1.83)	<0.001
Sibling with eczema	209 (20.4)	436 (11.3)	2.02 (1,69 to 2.43)	<0.001
Living in an urban area	631 (62.5)	2134 (56.0)	1.31 (1.14 to 1.51)	<0.001
Maternal medication during pregnancy	328 (32.2)	1066 (27.7)	1.24 (1.07 to 1.44)	0.005
Light sleeper at 6 months	321 (31.3)	1016 (26.2)	1.28 (1.10 to 1.49)	0.001
Troubled sleep at 6 months	150 (14.6)	434 (11.2)	1.36 (1.11 to 1.66)	0.003
3+ Awakenings per night	215 (21.8)	657 (17.7)	1.30 (1.09 to 1.54)	0.003
Male gender	574 (56.1)	1979 (51.1)	1.22 (1.06 to 1.40)	0.005
Dog at home	123 (11.9)	599 (15.3)	0.75 (0.61 to 0.92)	0.006
Bird at home	12 (1.2)	100 (2.6)	0.45 (0.25 to 0.82)	0.009
Age at introduction of fish				<0.001
0–2 Months	6 (0.7)	15 (0.4)	1.1 (0.4 to 2.9)	0.83
3–5 Months	194 (21.3)	750 (20.9)	0.7 (0.6 to 0.9)	0.003
6–8 Months	462 (50.8)	2142 (59.6)	0.6 (0.5 to 0.7)	<0.001
9–12 Months	248 (27.3)	689 (19.2)	1 (Ref)	Ref
Type of fat on bread				0.003
Dairy	674 (67.2)	2794 (72.3)	1 (Ref)	Ref
Margarine	280 (27.9)	878 (22.7)	1.32 (1.13 to 1.55)	0.001
Nil	49 (4.9)	191 (4.9)	1.06 (0.77 to 1.47)	0.71
Frequency of fish consumption				<0.001
3+ Times a week	54 (5.3)	195 (5.0)	1 (Ref)	Ref
1–3 Times a week	584 (57.0)	2400 (61.6)	0.88 (0.64 to 1.20)	0.42
1–3 Times a month	247 (24.1)	1038 (26.6)	0.86 (0.62 to 1.20)	0.37
A few times a year	57 (5.6)	156 (4.0)	1.32 (0.86 to 2.02)	0.20
Never	83 (8.1)	110 (2.8)	2.73 (1.80 to 4.13)	<0.001
Usually eats lean fish	723 (79.3)	2918 (82.5)	0.81 (0.68 to 0.97)	0.025
Frequency of lactic acid (yoghurt, fermented				<0.001
vegetables, etc) in diet				
3+ Times a week	497 (48.8)	1986 (51.1)	1 (Ref)	Ref
1–3 Times a week	257 (25.2)	1173 (30.2)	0.88 (0.75 to 1.04)	0.12
1–3 Times a month	110 (10.8)	419 (10.8)	1.05 (0.83 to 1.32)	0.69
A few times a year	29 (2.8)	131 (3.4)	0.89 (0.59 to 1.34)	0.56
Never	125 (12.3)	174 (4.5)	2.87 (2.24 to 3.69)	<0.001
Troubled sleep at 12 months	203 (19.7)	611 (15.6)	1.32 (1.11 to 1.58)	0.002

Variables with p between 0.01 and 0.05 were: maternal asthma, air pollution in neighbourhood, feeding difficulties in first week, breast feeding, maternal education, paternal education, light sleeper at 12 months.

Variables with p⩾0.05 were: number of siblings, sibling with asthma, cohabitation, maternal and paternal employment, type of housing, dampness or mould in the house, access to a summer cottage, lifestyle (leisure activities), regular maternity check-ups, maternal alcohol consumption during or after pregnancy, low birth weight, preterm birth, admission to neonatal ward, neonatal antibiotics, early introduction of cow’s milk (first week), temperament first week, weak sucking, vomiting, maternal and paternal smoking during pregnancy, maternal and paternal smoking after pregnancy, sleeping position, sleeping place, hours outdoors, sleeping indoors with outdoor clothes on, temperament at 6 months, colicky pains, appetite, dummy at 6 months, cohabitation, maternal return to work, moved house between 6 and 12 months, cat or rodent at home, age at introduction of cow’s milk, gluten, solids or eggs, type of fat used in cooking, vegetarianism in family, AD vitamin supplementation, appetite, vaccinations, antibiotics in first year, early antibiotic treatment, sleeping position, sleeping place, night awakenings, temperament at 12 months and dummy at 12 months.

#### Multivariable analysis

In the multivariable analysis, significant risk factors were maternal eczema, a sibling with eczema, a bird in the home, the introduction of fish before 9 months of age and cow’s milk allergy (table 3).

**Table 3:** Significant (at p<0.01) risk factors for eczema at 1 year of age in the multivariable analysis: the Infants of Western Sweden Study

Risk factor	OR (95% CI)	p Value
Maternal eczema	1.54 (1.30 to 1.84)	<0.001
Sibling with eczema	1.87 (1.50 to 2.33)	<0.001
Bird in the home	0.35 (0.17 to 0.75)	0.007
Introduction of fish before 9 months	0.76 (0.62 to 0.94)	0.009
Cow’s milk allergy	3.64 (2.35 to 5.64)	<0.001

Since breast feeding and smoking were non-significant (p>0.01) in the univariable analysis they were not included in the final multivariable model. However, since the effects of breast feeding and smoking on allergic disease are debated, we also carried out an analysis including these factors in the model. This confirmed that they were not statistically significant risk factors.

## DISCUSSION

This study shows that one in five infants have eczema during the first year of life and, as expected, there is a strong association with a family history of eczema. Furthermore, we found beneficial effects from introducing fish before 9 months of age, while short duration of breast feeding, keeping furry pets or the age at which milk or eggs were introduced did not affect the risk.

The KOALA Birth Cohort Study^19^ found a prevalence of eczema of 22.2% at 1 year. In the BAMSE study, the prevalence of eczema was 25.1% ever before the age of 2.^20^ Our study confirms the high prevalence of eczema during the first year of life, with a cumulative prevalence of 13.9% at 6 months and 20.9% at 12 months of age.

The prevalence of eczema was estimated using questionnaires sent to the parents, with questions on whether the infant had previous or current eczema at 1 year of age and did not require a doctor's diagnosis. This may lead to uncertainty about the exact prevalence, but as we found a prevalence similar to those in other cohort studies, we believe that the results are valid.

To further evaluate the validity of our study material, we have compared the prevalence of smoking during pregnancy in our study (9.5%) with recent statistics from the National Swedish Board of Health and Welfare which show that 10% of all pregnant women in western Sweden and in Sweden as a whole smoked in 2003.^21^ Since smoking is associated with several disadvantageous factors, we believe that the data from western Sweden are representative of the population.

The possibility of recall bias is always a limitation in questionnaire studies. However, as food data were collected at 6 and 12 months in this study, we do not think that recall bias is a major problem as the time intervals are relatively short.

Compared with data from Stockholm,^22^ more homes in western Sweden seem to have furry pets (15.4% vs 20%, respectively). This is probably because the BAMSE study recruited infants from central Stockholm, while our infants were from both urban and rural areas. In the western Sweden study, we found a higher prevalence of furry pets in rural areas.

There is consensus that sensitised children and children with symptoms of allergic disease should not have contact with furry pets.^23^ On the other hand, opinions differ as to whether furry pets in the local environment of a young child can act as a protective factor and help tolerance to develop,^7^ ^9^ ^10^ or whether animals are risk factors for the development of allergy in children in high-risk families. In our study, the initial protective effect of keeping a dog disappeared when adjusting for “atopic reasons” (manifest allergy or fear of allergy) for not keeping an animal. The protective effect of having a bird in the home may be due to reverse causation if non-atopic families keep more birds. However, some studies suggest that contact with feathers might prevent atopic disease.^24^^–^26 Since birds almost always are kept inside providing continuous indoor exposure, it may be that birds promote exposure to endotoxin. This might explain why we found a protective effect of bird keeping.

Previous primary preventive advice in Sweden recommended the delayed introduction of eggs and fish to infants with strong allergic heredity. Although this advice was abandoned in the autumn of 2002 because supportive evidence was lacking,^23^ we found that many parents waited to introduce eggs and fish. The results of our study show that outdated primary preventive advice which lacks evidence tends to be still followed by the general population. At the time of the study, allergy prevention advice placed no restrictions on the consumption of eggs or fish either in families at risk or in the rest of the population.

The results suggest that advice previously given at infant welfare clinics concerning the introduction of small portions of cereals and solids at 4 months of age is being followed, while cow’s milk-based formulas and weaning foods are introduced earlier by many parents. The WHO code advocating exclusive breast feeding up to 6 months of age has been recommended in Sweden since 2001. The early steep rise in the curve showing the introduction of cow’s milk (fig 2) could be explained by the parents (truthfully) counting every small helping of formula, for example during the neonatal period before the firm establishment of breast feeding.

In our study, the strongest risk factor for eczema was a family history of eczema and maternal eczema in particular. Our finding that a family history of eczema is a stronger risk factor than a family history of other atopic diseases is compatible with, for example, the ALSPAC study, where parental eczema was regarded as a better marker than parental asthma or hay fever.^27^ In fact, a Danish twin study found that genes accounted for 82% of individual susceptibility, while non-shared environmental factors accounted for 18%.^28^ Recently, it has been demonstrated that loss-of-function mutations in the filaggrin gene predispose to eczema. Filaggrin is a key protein of the epidermis that has an important role in the formation of the skin barrier. Loss of filaggrin function seems to play a role both in the pathogenesis of eczema and the subsequent progression of atopy.^29^ ^30^

In the multivariable analysis, we found that the introduction of fish before 9 months of age had a protective effect on eczema. This is compatible with the findings of Kull *et al*,^31^ who found that regular fish consumption in the first year of life was associated with a lower risk of eczema at 4 years of age. It has been suggested that a high ratio of omega-6 to omega-3 fatty acids, as is common in western diets, could contribute to the pathogenesis of allergic disease.^32^ The fact that fish is rich in omega-3 fatty acids could partly explain the effects found in this cohort. However, we found no influence of the type of fish ingested (lean/white or fat/oily) in this study, which is compatible with the CAPS study^33^ and recent data from Norway.^34^ The Norwegian study reported beneficial effects on the risk of developing allergic disease if the child ate fish, especially lean fish, but neither the CAPS nor the Norwegian study found that omega-3 fatty acids reduced the risk of allergic disease, including eczema. This makes it somewhat difficult to ascribe the effect to omega-3 fatty acids only.

Dairy products have also been suggested to have beneficial effects on allergic disease. In a study from the Netherlands, it has been shown that milk and butter consumption reduced the risk of asthma.^12^ However, we were not able to demonstrate any protective effects by dairy products in this study.

The results concerning a possible protective effect on eczema by breast feeding are contradictory. Kull *et al*^35^ found a protective effect by breast feeding on eczema at 4 years of age and protective effects have also been reported in other studies.^36^ ^37^ However, in this study we did not find that breast feeding had any protective effect on the occurrence of eczema. This is in line with the results of Belgian PIPO, the Dutch KOALA and the Australian CAPS studies.^9^ ^19^ ^38^

**What is already known on this topic**

- Eczema is common in infants.
- Heredity is a strong risk factor.

**What this study adds**

- The introduction of fish before 9 months of age reduces the risk of eczema.
- Breast feeding does not provide protection from eczema during the first year of life.

There are also a few studies that claim that prolonged breast feeding can increase the prevalence of eczema.^39^ ^40^ This may possibly be due to reverse causation, as infants with manifest eczema tend to be breast fed longer than other infants. However, the CAPS study found no significant association between the presence of eczema at 4 weeks and at 3 months and continued breastfeeding beyond those dates. Nor did we find any effects on the prevalence of eczema from parental smoking during or after pregnancy, which is in line with the PIPO study.^9^

In conclusion, we have found that one in five infants in western Sweden have eczema. In a multivariable analysis, we found large effects from heredity and no effect from the duration of breast feeding or the age at which milk or eggs were introduced, while protective effects from introducing fish before 9 months were found.

## References

[1] Williams H Stewart A Aït-Khaled N Worldwide variations in the prevalence of symptoms of atopic eczema in the International Study of Asthma and Allergies in Childhood. J Allergy Clin Immunol 1999;103:125–38 9893196 10.1016/s0091-6749(99)70536-1

[2] Sugiyama M Arakawa H Ozawa K Early-life risk factors for occurrence of atopic dermatitis during the first year. Pediatrics 2007;119:e716–23 17332188 10.1542/peds.2006-0893

[3] Åberg N Hesselmar B Åberg B Increase of asthma, allergic rhinitis and eczema in Swedish schoolchildren between 1979 and 1991. Clin Exp Allergy 1995;25:815–19 8564719 10.1111/j.1365-2222.1995.tb00023.x

[4] Asher MI Montefort S Björkstén B Worldwide time trends in the prevalence of symptoms of asthma, allergic rhinoconjunctivitis, and eczema in childhood: ISAAC Phases One and Three repeat multicountry cross-sectional surveys. Lancet 2006;368:733–43 16935684 10.1016/S0140-6736(06)69283-0

[5] Björksten B Sepp E Julge K Allergy development and the intestinal microflora during the first year of life. J Allergy Clin Immunol 2001;108:516–20 11590374 10.1067/mai.2001.118130

[6] Holt PG Current concepts in pulmonary immunology: regulation of primary and secondary T-cell responses to inhaled antigens. Eur Resp Rev 1996;6:128–35

[7] Hesselmar B Åberg N Åberg B Does early exposure to cat or dog protect against later allergy development? Clin Exp Allergy 1999;29:611–17 10231320 10.1046/j.1365-2222.1999.00534.x

[8] Melén E Wickman M Nordvall SL Influence of early and current environmental exposure factors on sensitization and outcome of asthma in pre-school children. Allergy 2001;56:646–52 11421923 10.1034/j.1398-9995.2001.00387.x

[9] Hagendorens MM Bridts CH Lauwers K Perinatal risk factors for sensitization, atopic dermatitis and wheezing during the first year of life (PIPO study). Clin Exp Allergy 2005;35:733–40 15969663 10.1111/j.1365-2222.2005.02254.x

[10] Linneberg A Simonsen JB Petersen J Differential effects of risk factors on infant wheeze and atopic dermatitis emphasize a different etiology. J Allergy Clin Immunol 2006;117:184–9 16387604 10.1016/j.jaci.2005.09.042

[11] Ludvigsson JF Moström M Ludvigsson J Exclusive breastfeeding and risk of atopic dermatitis in some 8300 infants. Pediatr Allergy Immunol 2005;16:201–8 15853948 10.1111/j.1399-3038.2005.00257.x

[12] Wijga AH Smit HA Kerkhof M Association of consumption of products containing milk fat with reduced asthma risk in pre-school children: the PIAMA birth cohort study. Thorax 2003;58:567–72 12832666 10.1136/thorax.58.7.567 PMC1746729

[13] Mihrshahi S Peat JK Marks GB Eighteen-month outcomes of house dust mite avoidance and dietary fatty acid modification in the Childhood Asthma Prevention Study (CAPS). J Allergy Clin Immunol 2003;111:162–8 12532113 10.1067/mai.2003.36

[14] Alm JS Swartz J Björkstén B An anthroposophic lifestyle and intestinal microflora in infancy. Pediatr Allergy Immunol 2002;13:402–11 12485315 10.1034/j.1399-3038.2002.01062.x

[15] Cochran WG Sampling techniques New York: Wiley, 1977

[16] Alm B Möllborg P Erdes L SIDS risk factors and factors associated with prone sleeping in Sweden. Arch Dis Child 2006;91:915–19 16464961 10.1136/adc.2005.088328 PMC2082937

[17] Alm B Erdes L Möllborg P Neonatal antibiotic treatment is a risk factor for early wheezing. Pediatrics 2008;121:697–702 18381533 10.1542/peds.2007-1232

[18] Norušis M SPSS 13.0 guide to data analysis Upper Saddle River, NJ: Prentice Hall, 2005

[19] Snijders BE Thijs C Kummeling I Breastfeeding and infant eczema in the first year of life in the KOALA birth cohort study: a risk period-specific analysis. Pediatrics 2007;119:e137–41 17200240 10.1542/peds.2006-0772

[20] Böhme M Lannerö E Wickman M Atopic dermatitis and concomitant disease patterns in children up to two years of age. Acta Derm Venereol 2002;82:98–103 12125961 10.1080/00015550252948112

[21] Socialstyrelsen Smoking habits among pregnant women and parents to small infants 2002 [*Tobaksvanor bland gravida och spädbarnsföräldrar 2002*] Stockholm: Socialstyrelsen (Swedish National Board of Health and Welfare), 2004

[22] Wickman M Melén E Berglind N Strategies for preventing wheezing and asthma in small children. Allergy 2003;58:742–7 12859552 10.1034/j.1398-9995.2003.00078.x

[23] Swedish Society of Paediatric Allergology (2006). Prevention - information om möjlighet att minska risken för astma och allergisk sjukdom hos barn under första levnadsåren [Prevention - information about possibilities to decrease the risk of asthma and allergic disease in infants and children during the first years of life].. http://www.barnallergisektionen.se/stenciler_nya06/a1_prevention.html.

[24] Nafstad P Nystad W Jaakkola JJ The use of a feather quilt, childhood asthma and allergic rhinitis: a prospective cohort study. Clin Exp Allergy 2002;32:1150–4 12190650 10.1046/j.1365-2745.2002.01415.x

[25] Ponsonby AL Kemp A Dwyer T Feather bedding and house dust mite sensitization and airway disease in childhood. J Clin Epidemiol 2002;55:556–62 12063097 10.1016/s0895-4356(01)00519-4

[26] Strachan D Carey IM Reduced risk of wheezing in children using feather pillows is confirmed. BMJ 1997;314:518 9056820 10.1136/bmj.314.7079.518 PMC2125974

[27] Wadonda-Kabondo N Sterne JA Golding J Association of parental eczema, hayfever, and asthma with atopic dermatitis in infancy: birth cohort study. Arch Dis Child 2004;89:917–21 15383434 10.1136/adc.2003.034033 PMC1719677

[28] Thomsen SF Ulrik CS Kyvik KO Importance of genetic factors in the etiology of atopic dermatitis: a twin study. Allergy Asthma Proc 2007;28:535–9 18034971 10.2500/aap2007.28.3041

[29] Palmer CN Irvine AD Terron-Kwiatkowski A Common loss-of-function variants of the epidermal barrier protein filaggrin are a major predisposing factor for atopic dermatitis. Nat Genet 2006;38:441–6 16550169 10.1038/ng1767

[30] Marenholz I Nickel R Ruschendorf F Filaggrin loss-of-function mutations predispose to phenotypes involved in the atopic march. J Allergy Clin Immunol 2006;118:866–71 17030239 10.1016/j.jaci.2006.07.026

[31] Kull I Bergström A Lilja G Fish consumption during the first year of life and development of allergic diseases during childhood. Allergy 2006;61:1009–15 16867056 10.1111/j.1398-9995.2006.01115.x

[32] Fogarty A Britton J The role of diet in the aetiology of asthma. Clin Exp Allergy 2000;30:615–27 10792352 10.1046/j.1365-2222.2000.00766.x

[33] Marks GB Mihrshahi S Kemp AS Prevention of asthma during the first 5 years of life: a randomized controlled trial. J Allergy Clin Immunol 2006;118:53–61 16815138 10.1016/j.jaci.2006.04.004

[34] Øien T Storrø O Johnsen R Fish and cod liver oil consumption during pregnancy and the first year of life and allergic diseases at 2 years of age. A prospective birth cohort study. Allergy 2008;63 (Suppl 88):53

[35] Kull I Böhme M Wahlgren CF Breast-feeding reduces the risk for childhood eczema. J Allergy Clin Immunol 2005;116:657–61 16159639 10.1016/j.jaci.2005.04.028

[36] Kramer MS Chalmers B Hodnett ED Promotion of Breastfeeding Intervention Trial (PROBIT): a randomized trial in the Republic of Belarus. JAMA 2001;285:413–20 11242425 10.1001/jama.285.4.413

[37] Laubereau B Brockow I Zirngibl A Effect of breast-feeding on the development of atopic dermatitis during the first 3 years of life--results from the GINI-birth cohort study. J Pediatr 2004;144:602–7 15126993 10.1016/j.jpeds.2003.12.029

[38] Mihrshahi S Ampon R Webb K The association between infant feeding practices and subsequent atopy among children with a family history of asthma. Clin Exp Allergy 2007;37:671–9 17456214 10.1111/j.1365-2222.2007.02696.x

[39] Bergmann RL Diepgen TL Kuss O Breastfeeding duration is a risk factor for atopic eczema. Clin Exp Allergy 2002;32:205–9 11929483 10.1046/j.1365-2222.2002.01274.x

[40] Pesonen M Kallio MJ Ranki A Prolonged exclusive breastfeeding is associated with increased atopic dermatitis: a prospective follow-up study of unselected healthy newborns from birth to age 20 years. Clin Exp Allergy 2006;36:1011–18 16911357 10.1111/j.1365-2222.2006.02526.x

